# Textual heritage and digital archives – the case of the Hyakugo Archive in Kyoto

**DOI:** 10.12688/openreseurope.16820.1

**Published:** 2023-12-20

**Authors:** Edoardo GERLINI

**Affiliations:** 1 Department of Asian and North African Studies, Ca' Foscari University of Venice, Venice, Veneto, 30123, Italy

**Keywords:** textual heritage, digital archive, Toji Hyakugo Archive, Kyoto, cultural heritage, premodern Japan, Maeda Tsunanori

## Abstract

What are the effects and significance of inscribing an archive or group of documents in a heritage list? In light of the positive effects of digital technology on archival science, should all archives and past documents be considered “heritage,” or are some more significant than others? What are the implications and benefits of a heritage archive? Is creating a digital database of a specific archive considered part of heritage conservation? Is the term "heritagization" or "heritage making" a synonym for preservation or conservation?

In this article, I will attempt to answer some of these questions from the point of view of premodern literature, drawing on recent researches in heritage studies, specifically in the subfield of "critical heritage studies. After briefly introducing the current state of heritage scholarship, I will present the definition of "textual heritage" that I developed during my most recent project. Secondly, to reflect on how the concept of textual heritage can affect our understanding of historical archives, I will present the case of the Hyakugo Archive of Toji Temple (Kyoto, Japan), a collection of 19,000 documents dating from the eight to the eighteenth century, which was inscribed on the UNESCO Memory of the World List in 2005 and has been fully digitized and made available to the public via the Internet. I will examine a particular historical event that occurred during the 17th century, which can be viewed as a re-birth of this archive as a cultural heritage and reflect on the implications of this event for the survival of the archive itself and its use today.

## Introduction

The rapid and exponential development of digital technologies in the first two decades of the twenty-first century has made it possible to access, share, and study premodern documents and texts in ways unimaginable before, opening the field to new scholarly approaches and methodologies commonly referred to as the "digital humanities". The digitalization and organization of documents into freely accessible internet databases has revolutionized the way in which researchers in the humanities, including philologists and historians, conduct their studies, leading to improved quantitative and qualitative results. The digitization of archives and ancient documents has also encouraged libraries and cultural institutions to open up their documentary resources, giving readers and students in remote or underdeveloped areas, as well as those not directly involved in scholarly activity fairer access to knowledge. This has led to the possibility of transcending physical, geographical, and political boundaries through the internet.

Many programs for the digitization of documents and archives, especially those funded by public organizations and governments, go to great lengths to demonstrate the positive impact that these initiatives have on society and local communities. The underlying concept is that equitable and transparent access to knowledge is critical for building inclusive, resilient, and better societies. This idea is based on the principle that everyone should have the right to explore, understand, and enjoy the cultural products of humanity, which should be understood as shared heritage. Also from a normative point of view, access to culture is now recognized as an integral part of human rights, as clearly stated in the Universal Declaration of Human Rights (Art. 27): “Everyone has the right freely to participate in the cultural life of the community, to enjoy the arts and to share in scientific advancement and its benefits.”

Equally connected to human rights and equitable access to culture is a concept that has gained popularity amongst scholars, media, and the public discourse surrounding culture in the past 30 years: "heritage.” The label "heritage” is today attributed to a wide range of items, including tangible ones like buildings, artworks, and natural landscapes, as well as intangible ones like songs, dances, plays, rites, and craftsmanship. In the late 20th century, the label of "culture" was not necessarily applied to the latter, such as old factories, agricultural techniques, and food. In particular, the notion of heritage, especially the intangible variable, has enabled local communities and ethnic minorities to strengthen their cultural identity by giving social and symbolic meanings to their traditions. In doing so, they have challenged the authorized paradigms of cultural evaluation and artistic appreciation, which have been largely hegemonic and Western-centric.

The combination of digital technologies and the preservation and accessibility of cultural heritage are receiving increasing attention from institutional actors ^1^. In 2003, UNESCO published a " Charter on the Preservation of the Digital Heritage," alongside the promotion of the well-known "UNESCO Convention for the safeguarding of the Intangible Cultural Heritage." In documents related to another important cultural program, Memory of the World, UNESCO also argues for the inclusion of an increasing number of types of digital documents in the category of "documentary heritage," for example, through a spin-off project called Software Heritage launched in 2018, which aims to preserve the source code of popular but outdated software as a specific kind of cultural heritage.

The incorporation of digital technologies and the theoretical notion of heritage into the humanities field appears to be imperative today in order to challenge and reassess traditional assumptions about culture, its representation, universal values, and the Western-centric viewpoint that influences the majority of academic disciplines in modern universities. What are the effects and significance of inscribing to an archive or a group of documents in a heritage list? In light of the positive effects of digital technology on archival science, should all archives and past documents be considered “heritage,” or are some more significant than others? What are the implications and benefits of the heritage archives? Is creating a digital database of a specific archive considered a part of heritage conservation? Is the term "heritagization" or "heritage making" a synonym for preservation or conservation?

In this article, I will attempt to answer some questions as a premodern literature scholar, utilizing my recent research in heritage studies, specifically in the subfield of "critical heritage studies. After briefly introducing the current state of heritage scholarship, I will present the definition of "textual heritage" that I developed during my most recent project. Secondly, to reflect on how the concept of textual heritage can affect our understanding of historical archives, I will present the case of the Hyakugo Archive of Toji Temple (Kyoto, Japan), a collection of 19,000 documents dating from the eighth to the eighteenth century, which was inscribed on the UNESCO Memory of the World List in 2005 and has been fully digitized and made available to the public via the Internet. I will examine a particular historical event that occurred during the 17th century, which can be viewed as the rebirth of this archive as a cultural heritage and reflect on the implications of this event for the survival of the archive itself and its use today.

Japan is an ideal example when discussing digital archives and cultural heritage, as it has continuously produced an extensive collection of documents, books, and manuscripts since the introduction of Chinese writing in the sixth century, which is currently undergoing a significant digitization process and is stored primarily in archives and libraries throughout the country. Japan is also important because it has played a central role in promoting the concept of intangible heritage, redefining the meaning of terms such as "authenticity" or "conservation," often in contrast to the Western-centric paradigms that dominated the heritage discourse in the 20th century.

Reflecting on how archives are preserved, managed, and utilized in Japan can provide a novel perspective for globally reconsidering the significance and function of cultural heritage in the digitalized world of the 21st century.

## Heritage studies and the “missing” textual heritage

The definition of "heritage" has undergone complex changes over the past three decades. The "UNESCO World Heritage Convention," which came into force in 1972, resulted in the emergence of a rich "heritage industry" ^2^ in the 1980s. This has led to mass tourism and economic exploitation of the listed cities and sites. The competition among countries to assert ownership over the most significant and rich cultural resources, a sort of "world cup of heritage," started, and "national heritage" became a favored way for governments to reinforce feelings of national identity and unity among their citizens.

However, especially since the 1990s and outside the institutionalized boundaries of UNESCO and other national and international agencies, cultural heritage has also emerged as a powerful tool in the hands of minorities and discriminated communities to demand social justice and civil rights ^3– 5^. This new idea of heritage “challenges its previous core function as a bedrock of monocultural nation-building projects, a continuation of elitist cultural canons, and as upholding Eurocentric cultural values” ^6^.

The rise of heritage in the academic world has encouraged and stimulated the birth of an interdisciplinary field known as "heritage studies", which crosses a wide range of disciplines such as archaeology, history, law, sociology, anthropology, geography, economics, management, and more. In the past two decades, theoretical definitions of "heritage" have increasingly rejected the belief that cultural properties are solely to be identified with their material components. Especially scholars embracing the paradigm of “critical heritage studies” support the “idea of heritage not so much as a ‘thing’, but as a cultural and social process, which engages with acts of remembering that work to create ways to understand and engage with the present.” ^7^. Another point that is often stressed is that “alongside any intrinsic value heritage may have, ultimately meaning resides in the “intangible” relationships it provides between people and things.” ^8^

Even though this interpretation of cultural heritage may be too biased toward the ‘intangible’ side of culture, it also reflects a change in institutional discourse, as it has already taken place in the text of official conventions and declarations. The "Convention on the Value of Cultural Heritage for Society" (also referred to as the Faro Convention), which was adopted by the Council of Europe in 2005, provides a clear explanation.

The Faro Convention emphasizes the important aspects of heritage, as they relate to human rights and democracy. It promotes a wider understanding of heritage and its relationship to communities and society. The Convention encourages us to recognize that objects and places are not, in themselves, what is important about cultural heritage. They are important because of the meanings and uses that people attach to them and the values they represent. ( Council of Europe homepage).

The focus of heritage scholars has transitioned from places and monuments to people and communities and from things to practices, and a distinctive aspect of critical heritage studies is indeed a keen engagement with the political implications of defining and managing heritage and culture. This shift is evident, for example, in the following statement from the editors’s preface to the recent *A Companion to Heritage Studies* (Wiley Blackwell).

Starting from a position of seeing “heritage” as a mental construct that attributes “significance” to certain places, artifacts, and forms of behavior from the past through processes that are essentially political, we see heritage conservation not merely as a technical or managerial matter but as cultural practice, a form of cultural politics. ^9^

This connection between heritage and cultural politics has recently been emphasized by declarations of cultural diversity and human rights. For instance, Article 7 of the UNESCO Universal Declaration of Cultural Diversity, titled “Cultural heritage as the wellspring of creativity,” states the following:

Creation draws on the roots of cultural traditions, but flourishes in contact with other cultures. For this reason, heritage in all its forms must be preserved, enhanced, and handed on to future generations as a record of human experience and aspirations to foster creativity in all its diversity and to inspire genuine dialogue among cultures. (UNESCO, 2001)

The imperative to preserve heritage in all forms should not be confused with “any kind of heritage” or “heritage of everyone”. Traditional rituals or customs that infringe upon human rights, although they may be perceived as cultural heritage by certain communities, do not necessarily align with the values espoused by UNESCO and, more generally, by Western democracies. Consequently, it is impossible to assign the same space, importance, and social value to each and every “heritage” or “memory.” The emergence of “dissonant” or “difficult” heritage is simply a natural outcome of enabling multiple agents to utilize the concept. ^10^

This difficulty was the cause of the suspension of UNESCO's “Memory of the World” (MoW) Programme for a few years. In 2015, the Japanese government threatened to stop funding UNESCO due to the inclusion of the "Documents of Nanjing Massacre" in the MoW. These documents contain information on war crimes committed by the Japanese army during their occupation in China. From the Japanese government’s standpoint, the inclusion of these documents in the register harmed Japan's reputation as an impartial member of the international community. ^11^ In 2016, a request to include the “Voices of comfort women,” namely the testimonies of Korean women forced to work as prostitutes in Japanese army brothels, met with similar opposition from Japan. ^12^

 It has been observed that governments are utilizing the Memory of the World Programme for political gain and recognition of past atrocities on an international level ^13^. This trend has likely developed because of the program's focus on historical documents, which can be used to legitimize different versions of history depending on the reader's perspective and nationality. As a result, the program has become increasingly politicized, and therefore criticized for the risk of becoming a tool to promote specific interests and agendas rather than universal and democratic values. ^14^

In addition to the MoW example, scholars agree that heritage today is no longer just a label for monuments of the past that have "outstanding universal value," as outlined in the 1972 World Heritage Convention. Instead, they should be viewed as a dynamic and multifaceted social, cultural, and political practice. It is a “verb” ^15^, that can change in significance and value over time, varying across societies and perspectives depending on its use.

## Texts and archives as “heritage”

Given the previous reflection on heritage, what occurs when we reconsider an archive or collection of documents as cultural heritage? Is it feasible to reimagine archives and historical documents as a "social practice" or as a "verb"? Even if we acknowledge that the social, cultural, and political process of re-evaluation pursued in the present, known as "heritagization," is what truly makes something from the past a heritage, it seems that both the definitions of heritage given by official institutions such as UNESCO and by numerous scholars of heritage studies do not entirely apply to textual sources, documents, archives, or literary works. This is likely why, until now, few humanities scholars have directly engaged in heritage studies and why terms such as "textual heritage" or "literary heritage" have been used with nonchalance to highlight the value of specific documents or works from the past, or a literary canon, without a clear connotation within the heritage field. On the other hand, theories about heritage appear to have had minimal or no influence on the work of philologists and paleographers, who concentrated on the meticulous examination of ancient manuscripts.

Thanks to an EU-funded fellowship under the “Marie Sklodowska-Curie Action” programme, which I pursued from 2018 to 2021, I proposed to better define the boundaries and characteristics of what might be called "textual heritage" and to suggest new paths in the interdisciplinary dialogue between the humanities and heritage studies, with a particular focus on Japanese textual resources. ^16^ This project involves re-examining the concept of "text." Texts are a particular type of cultural products that can be replicated across various media without necessarily losing information or meaning. A text, particularly those inscribed on stone, can remain buried underground for hundreds or thousands of years. It may lie dormant and then be resurrected, ready for reading and analysis, owing to the efforts of archaeologists and philologists, thereby once again becoming part of our heritage. This was the case with the renowned Rosetta Stone.

What other heritage types possess this potential? What kind of heritage is "textual heritage"? This question revolves around reflection on the role and significance of digitizing documents and archives, and prompts consideration of the ultimate source of "heritage value," whether in the material object itself or in its content. The aspiration of archivists and scholars has always been to preserve and pass on any document, archive, or information from the past that could be utilized for novel research and inquiries to future generations. However, this goal remains virtually unattainable. A sad reality is that a large number of physical documents are disposed of due to the limitations of physical space and economic constraints faced by many libraries and archives on a daily basis.

Recent technological advancements may provide a solution to this conundrum by enabling the preservation of digital copies of every document produced by humanity in the last few centuries. Digitization practices improved the quality of digital copies of manuscripts or books to a level of detail unimaginable just a few years ago ^17^. Not only has the resolution of the scans improved, but we also now have new techniques that gather data about the physical characteristics of the medium, such as transparency, reflections, erased inscriptions, chemical composition of the ink, and organic residues on the paper. It is difficult to determine whether these intricately detailed digital duplicates will ever surpass the value of the original. Physical items retain the potential to allow for new, as yet undiscovered methods of analysis. They also possess a distinct vibe of genuine authenticity and uniqueness, which cannot be reproduced digitally.

This is especially true for works of art such as paintings or statues. But what about texts? If we view text solely as the written contents of a book, manuscript, or document, it is evident that it constitutes a unique form of cultural product that is quite distinct from a painting, a building, or even a song or a performance. Nobody can create an exact replica of Leonardo's Mona Lisa today. Even an apparently flawless copy will have some material differences that become apparent at the microscopic level. Consequently, any Mona Lisa copy will remain just that – a copy or fake. In contrast, copying a text meticulously – even a lengthy one like a novel – will not be considered a forgery, as the contents will be identical to the original.

This is even truer in the case of modern printed publications, which are, by definition, copies. Usually, readers do not need to read a novelist's handwritten drafts in order to feel a more authentic or correct "original.” This is also because today, the original text of a novel or any other text is primarily written on a computer. In other words, anyone who can read and write can produce a perfect copy of a document or book with just a pen, sufficient paper, and time. The case of the manuscripts is apparently different. As long as I am able to read it, a copy can always be produced. Moreover, if a sufficiently high-quality scan or photo of the manuscript exists, it would be possible to read it with nearly the same accuracy even if the original were lost. Therefore, excluding the more truly "artistic" and analogical aspects of a document - as in the case of calligraphy in a Japanese or Chinese ink painting or scroll - we can say that in most cases a written text is just a sequence, a chain of signifiers (namely the characters) lined up on a surface. By recognizing each individual character, one can faithfully copy any text without loss of information, even if it is written in an unknown language.

I believe this is why the management and sharing of textual products differs from those of heritage sites or intangible performances. The ways in which a text can be accessed are virtually limitless, as the number of digital copies that can be made is boundless. Therefore, texts are not subject to spatial and temporal constraints, such as those experienced by the historical centers of cities such as Venice or Kyoto, which are crowded with tourists. Independent of the number of copies of the text produced or the number of people currently reading it, either in print or online, the original owners will always retain their ability to access the text.

Regarding texts, I contend that their relationship with the digital dimension runs deeper than that of other cultural products. This connection is embedded in the word "text" itself. We frequently overlook that the term "text" originates from the Latin "tèxtum" and the verb "tèxtere,” meaning "fabric” and "to weave,” respectively. Invited to reflect on the concept of textual heritage in Japan, scholar Inaga Shigemi proposed the loom as a metaphor for text production and its inherent "digital" nature. According to Shigemi, “the loom is the earliest digital tool created by humans.” ^18^ This statement implies that the operator's identity holds no significance in weaving, as following the digital warp and weft pattern will produce identical fabrics regardless. Inaga extends his concept of the permeability of digital text and the natural world by proposing that the patterns on the skins of tigers and zebras, as well as the webs spun by spiders, may be deliberate outcomes of the information encoded in their DNA - a type of “digital text ( *denshi tekusuto*).” ^19^

A part how much we stretch the definition and range of the word "text," it is undeniable that it is closely linked to the concept of "digital." Digital resources, such as audio and video contents viewed and heard on our computers and smartphones, consist of a complex sequence of data - strings of “0s” and “1s” that are decoded according to specific rules. These data are written on digital memory or streamed through the Internet, and are ultimately executed or read by our devices. Moreover, software source codes are simply texts comprising alphanumeric characters organized in a programming language that computers can understand.

Therefore, as Inaga suggests, the creation of "text" stands out as one of the earliest and most significant digital innovations in human history. The production and dissemination of texts through duplication began well before the advent of computers, operating under rules distinct from those governing other forms of analog cultural products. Therefore, it is essential to establish a clear definition for "textual heritage" to address compatibility problems between texts and current heritage definitions. Reimagining the text as a native digital product compels us to recognize "textual heritage" as an implicit aspect of "digital heritage."

As previously mentioned, the UNESCO Memory of the World Programme includes a specific initiative titled "Software Heritage" that aims to preserve software codes as a valuable series of digital texts. In contrast, the textual heritage referred to here encompasses both analog and digital textual sources.

Textual heritage, as a means of examining the utilization and recreation of texts across time, can facilitate connections between disciplines that analyze written works, from philology to historiography to paleography, and computer science and software-related fields. This approach offers fresh and innovative insights into the evolution of textual cultures, the impact of texts on society, and the changing relationships between human beings and written works over time and across geographical locations.

If we attempt to tentatively define textual heritage, the following can be asserted:

Textual heritage refers to both tangible texts that have been transmitted from the past and intangible practices that were performed with and around them. For tangible texts, we refer to both physical embodiments, such as books, manuscripts, and steles, as well as digital backups, such as USB drives, containing the immaterial content (the code) inscribed on them. On the other hand, the intangible cultural practices of textual heritage refer to activities such as reading, writing, copying, collecting, translating, annotating, teaching, correcting, performing, collating, restoring, and more.
Textual heritage pertains to both the transmission of the physical existence of written material, as well as the intangible knowledge required to utilize and replicate it. This knowledge creates new values, meanings, and interpretations in the text itself.

While my definition is influenced by critical heritage studies' emphasis on the intangible nature of heritage, my intention is also to assert the vital role of tangible texts in the cultural process of producing and reproducing textual works.

To put it simply, there cannot be "intangible" cultural practices of "reading" if we don't have a book to read. Likewise, books cannot exist without someone possessing intangible knowledge to write them. Thus, tangible texts and intangible knowledge are both integral to textual heritage, and they affect each other in a spiral. Alternatively, if you prefer it, written materials embody the diverse intangible elements of reading and writing while simultaneously informing and constraining new developments in the intangible practices around them.

In a previous paper ^20^, I examined the incorporation of intangible heritage into physical artifacts using Ian Hodder’s entanglement theory ^21, 22^. I want to emphasize that the concept of textual heritage has the potential to stimulate further reflections on the essence of heritage more broadly, not just within literary studies. This could fuel discussions on how texts, documents, and archives function as cultural heritage.

## UNESCO Memory of the World and the documentary heritage

Once we have tentatively defined what constitutes "textual heritage," we can then ask the question: what occurs when an archive or group of historical documents is designated as a textual heritage? What issues arise during the process of "heritagization" in relation to archival sources?

The Memory of the World (MoW) program I talked about before was designed to safeguard and raise awareness of the world's "documentary heritage," which is defined by UNESCO as follows:

3.1.2. Documentary heritage comprises single documents – or groups of documents – of significant and enduring value to a community, culture, country, or humanity generally, and whose deterioration or loss would be a harmful impoverishment. The significance of this heritage may only become clear with the passage of time. ^23^

According to UNESCO, not every text of the past deserves the title of “documentary heritage”: only those with a “significant and enduring value.”

As previously stated, scholars often lament the lack and loss of archival documents and old writings, and are equally fascinated by the idea of preserving, cataloging, and passing on to future generations any kind of documentation about the past. This is because seemingly insignificant details, such as an advertisement page found in a magazine, can provide valuable insights into the values, goals, and cultural norms of previous societies. At present, it is unclear whether this scenario will materialize because of the digitization and management of big data. Nonetheless, it is crucial to emphasize that preserving data and texts does not automatically confer heritage status on them. This is also the stance of the MoW. It could be argued that every line written by a human is automatically part of the world's textual heritage, but this perspective fails to elucidate how cultural products are chosen, assessed, and disposed of. Heritagization entails more than simple preservation, as described by Rodney Harrison.

Heritage is not a passive process of simply preserving things from the past that remain but an active process of assembling a series of objects, places, and practices that we choose to hold up as a mirror to the present, associated with a particular set of values that we wish to take with us into the future. ^24^

Making something part of a heritage involves incorporating a set of knowledge, items, places, or customs into the living memory of a specific community, whether that community is local, national, or international. Simply recording a historical fact does not automatically make it present or active in the shared memory of a community. Past and memory are distinct, as are history and heritage, as often noted by David Lowenthal, a founding figure in the field of heritage studies. “Heritage is not history: heritage is what people make of their history to make themselves feel good” ^25^; “History remains remote; personal immediacy is a heritage hallmark” ^26^. The universalistic approach to heritage that characterized both the World Heritage Convention of 1972 and the Memory of the World Program eventually led to conflicts and criticism. The tension between memory and history is one of the more problematic aspects of the MoW programme, as its guidelines somehow conflate the words "memory" – typically individual and fragmentary – with "document" or "record" – that are supposed to be objective and historically reliable. When defining "memory institutions,” the General Guidelines state:

3.1.3 Memory institutions may include but are not limited to archives, libraries, museums and other educational, cultural and research organizations. ^27^

As Lowenthal notes, heritage, memory, and historical documents are not the same thing because they serve very different functions. While memory resides in the minds of living people, a document has the potential to remain unnoticed for centuries on a bookshelf or hidden in a cave. Heritage is a multifaceted social phenomenon that takes place in the present but relies on the past as evidence of its authenticity. The inclusion of an archive in a heritage list is primarily a political decision, similar to any other type of heritage listing. It occurs when a community - be it a small group of individuals or even a single individual- takes possession of a particular document or a group of documents as a whole and charges them with symbolic capital, usually for the purposes of self-legitimation and identity-building.

As one of the principal agencies of the United Nations, UNESCO's political goals are explicitly stated in its conventions and declarations. Regarding the Memory of the World Programme, its primary "political" objective is:

Underlining the importance of documentary heritage to promote the sharing of knowledge for greater understanding and dialogue, in order to promote peace and respect for freedom, democracy, human rights, and dignity ^28^

One could argue that this approach to memory and documentary heritage's role in solidifying international cooperation conflicts with the fact that heritage is also a tool for managing and utilizing the past to accommodate the present needs of different national and local communities. It is worth noting that this is a nuanced issue that requires consideration of the various perspectives involved. Heritage practices in certain communities or countries can lead to conflicts with others. This is particularly evident regarding the Memory of the World, as illustrated by past incidents concerning the “Documents of the Nanjing Massacre” and the “Voices of Comfort Women.” The nomination of Hashima Island as a World Heritage site in 2015, a major site for coal mining and shipbuilding during Japan's Meiji Industrial Revolution, received similar criticism because many Koreans were forced to work there under semi-slavery conditions.

The solution to this conundrum might be to accept the idea that heritage is not universal, that it can generate conflict, and that it has been used and understood differently in different places and at different times. However, it remains an essential aspect of the cultural identity of individuals and communities.

## Making textual heritage in the past: the Hyakugo archive of Tōji temple

Even if the word "heritage" in its contemporary sense is a product of Western civilization, directly linked to the history of the formation of the modern nation-state in the late 19th century, the political, cultural, and social processes that we now define as "heritagization" have a much longer history. As David C. Harvey has argued perceptively, “heritage has always been with us and has always been produced by people according to their contemporary concerns and experiences.” ^29^ According to Harvey, it is therefore possible to create a “history of heritage […] by producing a context-rich account of heritage as a process or a human condition rather than as a single movement or personal project. […] Heritage resides in the here and now – whenever and wherever that here and now happens to be. “ ^30^

In the case study I will present in the following pages, the "here and now" is 17th-century Japan, and the object of heritagization is the archive known as the Hyakugō Archive of Toji Temple ( *Tōji hyakugō monjo*, in Japanese). This archive was inscribed on UNESCO's Memory of the World list in 2015, but even before that, it was the subject of a long process of assessment, preservation, restoration, digitization, and dissemination, which I think can be collectively called heritagization. I focus on one particular episode that gave the archive its actual name: the One Hundred Boxes ( *hyakugo*) archive.

The Hyakugo Archive is a collection of nearly 19,000 documents (consisting of 25,000 items), originally stored at Tōji, a Buddhist temple in southwestern Kyoto. The documents span approximately 1,000 years, from the eighth to the 18th centuries, with the bulk of the collection dating from the 14th and 15th centuries. Today, the collection is owned by the Kyoto Prefectural Government, which purchased it from Tōji Temple in 1967 and housed it in a storage facility of the Kyoto Institute, Library and Archives. The Hyakugo Archive is one of the most important historical sources for the study of legal and administrative systems in medieval Japan, particularly in relation to the economic power of Buddhist temples. Because of its historical value, the Japanese government declared it a national treasure in 1997. The inscription in the Memory of the World Register in 2015 sanctioned the importance of this archive on an international level and acknowledged the quality of the digitization process that took place in the preceding years.

Most of the documents in the Hyakugo archive are written on Japanese paper (washi) and inscribed with India ink. Although washi is known to be a very durable material, it is made from the fibers of local plants such as the gampi tree, the mitsumata shrub, or the paper mulberry bush (kōzo); when the documents were acquired by the Kyoto Prefectural Government, approximately 9,000 required urgent restoration, which took seven years and was completed in 1973. At the same time, archivists at the Kyoto Institute, Library, and Archives spent about ten years cataloging the entire collection (the complete five-volume catalog was published in 1976). After the Japanese government designated the collection as an Important Cultural Property in 1980, another 1,000 or so documents were restored. The remaining 9,000 documents were left untouched at the time of acquisition ^31^. From 1980 to 1983, the entire collection was photographed in 80,000 microfilm cuts, then printed, and archived in 485 photo albums that were used for daily consultation. Finally, in February 2014, the digitization of the entire collection was completed, and all of the more than 80,000 cuts were published online on the "Hyakugo Archives WEB" site under a "Creative Commons Attribution 2.1 Japan License" ( CC BY 2.1 JP).

The name of the collection, "One Hundred Boxes (hyakugō) of Documents," comes from the number of paulownia wooden boxes in which the documents were stored at Tōji Temple. The story of these boxes is closely tied to the survival of this collection, and I argue that it can be seen as an important episode - if not the first - of "heritagization" in the history of this archive. This story is not only relevant to archivists, as it is intertwined with the history of medieval Japan and its administrative and political systems, as well as the changing values and attitudes toward cultural and written artifacts.

As noted above, the core of the archive consists of certificates attesting to the Tōji's ownership of lands and estates, as well as other certificates used in the day-to-day management of various properties of the temple. According to the so-called *shōen* (manorial) system introduced in the eighth century, temples and shrines, as well as members of the aristocracy, had the right to privately own large estates scattered throughout the country that were basically tax-free and self-governed. This system of landownership was guaranteed by the *ritsuryō* legal codes, which were based on the issuance and possession of documents regularly used to certify the ownership of a specific property ^32^. High priests at Tōji preserved these documents in boxes called *tefubako*, which were used daily. As local feudal lords rose to power in the 15th and 16th centuries and with the beginning of the new unified administration of the Tokugawa shoguns in the 17th century, the *shōen* system ultimately declined, and all documents related to *ritsuryō* and estate administration suddenly became useless for claiming rights and privileges ^33, 34^.

At this time, a new way of using and valuing these documents began, as they were in danger of being forgotten and lost due to their lack of practical value. In pacified Japan of the 17th century, feudal lords loyal to the Tokugawa shogunate gained power, wealth, and land to govern. Consequently, they began to develop an interest in the study of letters and history, both for personal pleasure and as a way to acquire symbolic capital from their knowledge of the past. Many so-called *daimyo* (regional lords), together with the central government, actively promoted the development of Confucianism and the compilation of historical compendia, becoming sophisticated literati and patrons of culture. One such daimyo was Maeda Tsunanori (1643–1724), the fifth daimyo of the Kaga domain in northwestern Japan (present-day Kanazawa Prefecture), one of the richest regions owing to the presence of gold mines and other resources. Tsunanori sent his retainers to various parts of the country in search of books and personally borrowed a portion of the Tōji archives for his own consultation, partially copying them and compiling a catalog.

Upon returning to Tōji in 1685, Tsunanori donated 94 boxes, originally thought to be 100, of paulownia wood to the temple. The purpose of this donation was to safeguard documents from damages inflicted by time and bugs. They were called a "block" (katamari) by archivist and scholar Uejima Tamotsu because they helped keep the archive untouched and in its original shape since medieval times. Indeed, these boxes have protected the collection for over three centuries, closely guarded in the treasure chamber of the Tōji Temple until they were acquired by Kyoto Prefecture ^35^. During the 1970s, recataloging the collection, the archivists at the Kyoto Archive adhered to the original document organization within each box. It was evident that the documents were grouped together based on clear rules, rather than being randomly added to boxes one by one ^36^. The name of each box, which follows the two phonetic alphabets hiragana and katakana, and the sinograph kyō (capital city), is retained in the new catalog. This was a way of giving importance not only to the documents themselves, but also to the way in which they were managed and catalogued in pre-modern times, giving the macro-textual structure–the archive–a particular historical value *per se.*

Even in Tsunanori's time when those who had access to the archive were privileged elites, we can argue that Tsunanori himself looked at the archive as a whole and recognized in it a new meaning and value independent of the practical use the documents had had in earlier times. Although Tsunanori's purpose has almost nothing to do with the main goals of UNESCO's Memory of the World Programme, that is, international cooperation and human rights, I argue that the decision to donate the boxes to the temple in order to preserve this resource, as well as the re-evaluation and appreciation of outdated, useless documents, can be seen as an early example of heritagization, one of those pages of the "history of heritage" theorized by David Harvey. Tsunanori's selfless efforts to protect the archive allowed it to become, three centuries later, a "cultural heritage of the world" in the sense of UNESCO. The survival of the archive was not accidental or obvious, but the result of a reflection on the value of the past and its embodiment in written records and of an active preservation effort pursued by cultural and political elites. In other words, the cultural and social practice of meaning-making as it is theorized by scholars of critical heritage studies today. It is not clear whether Tsunanori donated the boxes with a view toward some kind of personal gain, such as the support of the still powerful Buddhist clerical class. However, it is undeniable that his donation made it easier for the monks of the Tōji Temple to take care of this tangible textual heritage. At the same time, this symbolic attribution of meaning and value, this attempt to preserve and care for the physical embodiments of the texts - as well as the process of borrowing, copying, and cataloging them - can be understood as an example of textual heritage, as I proposed in the definition above, namely, an intangible cultural practice that revolves around and upon texts.

This does not mean that in Tsunanori's actions we find the same ideals of "heritage" that we find today in UNESCO's conventions, such as "to promote universal access to documentary heritage" or "to increase worldwide awareness of the existence and importance of documentary heritage and thereby to promote dialogue and mutual understanding among peoples and cultures". ^37^ At the same time, it cannot be denied that Tsunanori's effort was a deliberate act of recognizing and transmitting the culture and memory of the past, just for the sake of future generations - not for Tsunanori's heirs or relatives - a fact that partially corresponds to the first objective of the MoW: ‘to facilitate preservation, by the most appropriate techniques, of the world’s past, present, and future documentary heritage.’ ^38^

## New value for useless texts: the prohibition order by Oda Nobunaga

**Figure 1.**
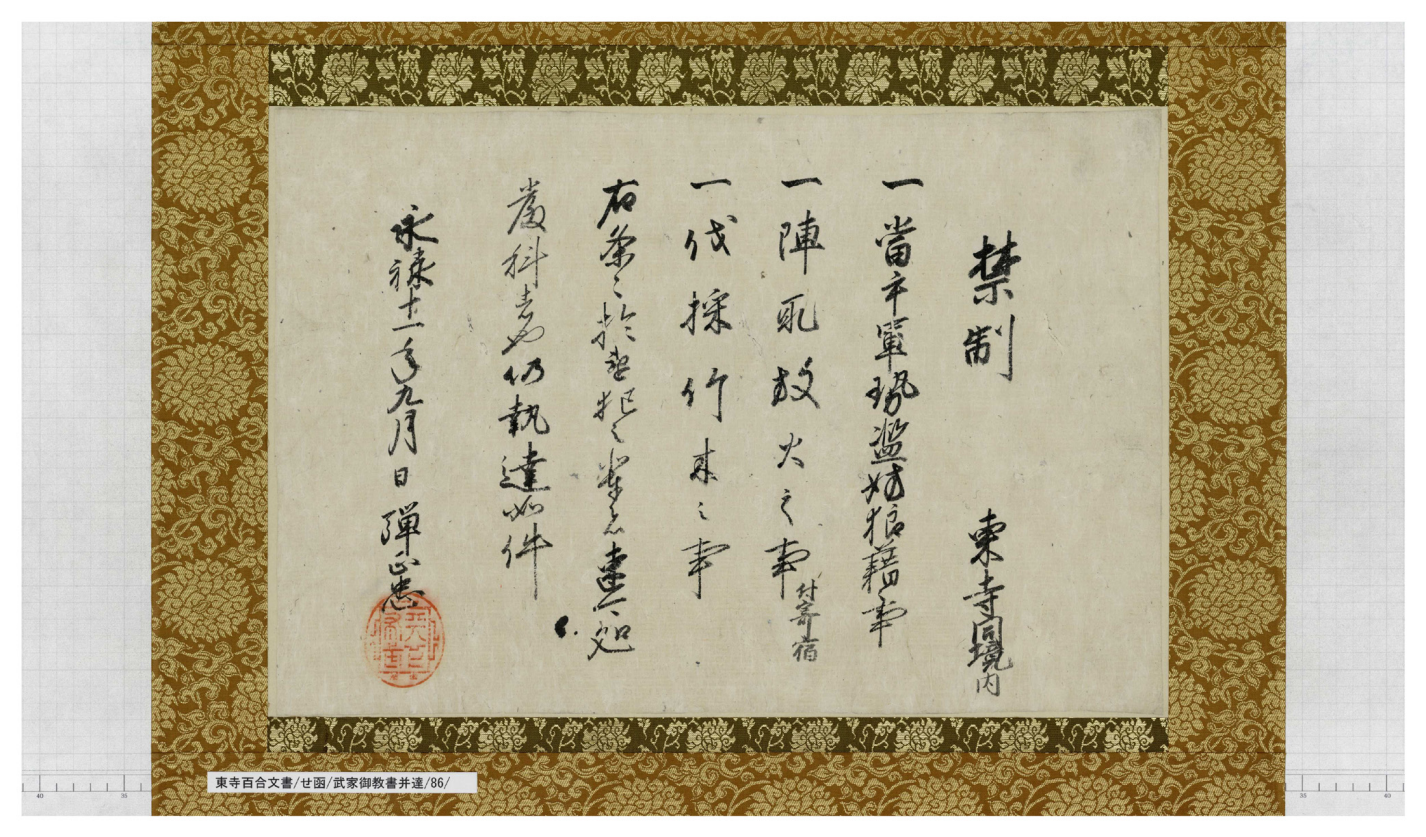
Box SE (Hiragana)/Documents for the education of samurai families/86. “Prohibition order by Oda Nobunaga.” This image is taken from the Toji Hyakugo Archives website, operated by the Kyoto Institute, Library and Archives. Available online at http://hyakugo.pref.kyoto.lg.jp/contents/detail.php?id=28572&p=2. This image is under a CC BY 2.1 JP license.

There is not enough space here to discuss which documents Tsunanori Maeda was particularly interested in and why he finally sent the wooden boxes to the Tōji Temple. I want to emphasize how the processes of heritagization affect the survival and value of certain historical texts. I will give just one example of how one of the documents in the Hyakugo Archive acquired a specific symbolic and economic value, not only, one might assume, in Tsunanori's time, but even in today's antiquarian book market. One of the documents of the Hyakugo Archives often displayed at exhibitions is a prohibition edict issued by Oda Nobunaga in the ninth month of the 11th year of the Eiroku era (CE 1568) ( Figure 1).

Oda Nobunaga (1534–1582) was one of the most important warlords who rose to power at the end of the 16th century and contributed to the reunification of Japan, which was completed by Tokugawa Ieyasu a few decades later. This document was written in the year Nobunaga first entered Kyoto with his army and contained three orders he gave to Tōji Temple (the second, third, and fourth lines from the right, respectively): prohibitions against assembling armies or occupying land, causing disturbances or setting fires, and cutting down trees.

These orders were intended to prevent any opposition from powerful temples in Kyoto during Nobunaga's occupation, and their validity was intended to be temporary. In any case, after Nobunaga's death in 1582, they had obviously lost any effectiveness. From a documentary point of view, this document is not particularly meaningful, as this type of order was regularly issued by feudal lords and Nobunaga himself signed many other documents addressed to various temples in Kyoto on the same occasion. It also does not provide any useful information or other details about the rise of Nobunaga. In other words, the historical value of this document is not so remarkable. Nevertheless, this document has been carefully preserved and is regularly displayed in exhibitions today, simply because it was issued and signed by Nobunaga, one of the most well-known historical figures in medieval Japan. Thus, a relatively trivial historical resource takes on a different meaning and value by being accepted as documentary heritage. People today, but also in the past, are fascinated by the fact that this document shows the authentic handwriting of one of the most powerful warlords who participated in shaping early modern Japan. Particularly appreciated is the presence, in the lower left corner of the page, of the vermilion seal with the characters *Tenka fubu* 天下布武, literally "to bring the martial (virtues) to the world," a sort of motto Nobunaga used when he began his military campaign to conquer Japan.

Visitors to today's exhibitions engage with Japan's past through this manuscript, enjoying the sight of Nobunaga's brush and seal. However, we can assume that literati in early modern Japan, such as Maeda Tsunenori, also enjoyed a similar kind of "fetishistic" appreciation. This process of imagining one's past through a physical object or remnant is precisely what heritage scholars refer to as heritage. It is also interesting to note that this type of symbolic value has always had an economic counterpart. If we take as a measure the prices at which Nobunaga's writings are usually sold at auctions or antique book fairs in Japan, we can estimate that this single page, if sold, could fetch no less than five million yen (about $45,000).

This is to say that the vast collection of the Hyakugō Archive not only has inestimable historical value, but also has been and will continue to be the object of processes of valorization and meaning-making that can be better explained and understood through the theoretical category of heritage, especially with the subcategory of "textual heritage" that I theorized earlier.

## Textual heritage today: the “Hyakugo archive WEB”

The inclusion of the Hyakugo Archive in the MoW Register in 2015 did not drastically change the way the archive itself is managed and preserved. As explained by Uejima ^39^, who was directly involved in the acquisition and cataloging process at the Kyoto Archive, the new catalogues, microfilms, and even the complete publication through an online database were completed before the UNESCO nomination and independently of the plan to propose it for inscription.

**Figure 2.**
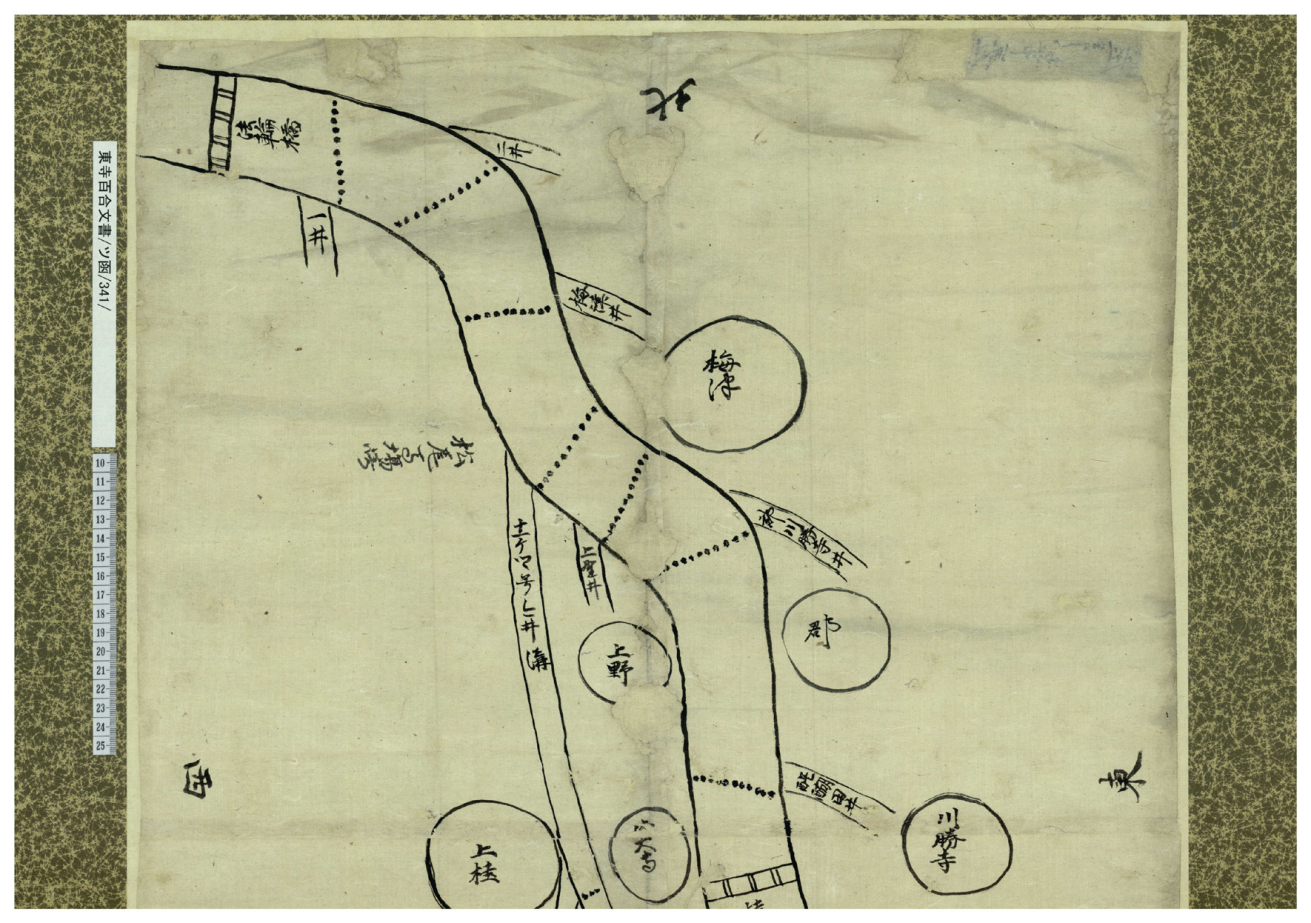
Box TSU(Katakana)/341, “Diagram of the use of water of the Katsura river in Yamashiro-guni”. This image is taken from the Toji Hyakugo Archives website, operated by the Kyoto Institute, Library and Archives. Available online at http://hyakugo.pref.kyoto.lg.jp/contents/detail.php?id=7340

Perhaps UNESCO's designation of the archive as a "world" documentary heritage has further encouraged the archive's curators to make its contents available to the public. In an effort that might be called "public archival science," the "Hyakugo Archives WEB" site proposes a new story about a single document from the collection every few months. It also suggests fieldwork on temple properties, both in Kyoto and in the countryside, to follow the documents and maps in the database ( Figure 2) ^40^. In another "story,” the site provides basic explanations on how manuscripts were protected from moisture and insects in medieval Japan. With a little delay, the stories are also translated into English to meet a larger international public.

Digitizing an archive, making it available on the Internet, retrieving individual documents to provide explanations of how people lived in the past are all cultural and social, and in a sense, political practices that allow people to make sense of the past, and in this sense can be called "(textual) heritage.” People who access the website and read these stories, as well as viewing the scans of the manuscripts, are given the opportunity to rethink or reinforce their cultural identity to negotiate a relationship with the past. This is true, of course, first and foremost for Kyoto residents or the Japanese in general, but also for anyone who accesses the site.

Even though donating wooden boxes and scanning documents seem to be two completely different practices, both Tsunanori and the Kyoto Prefectural Archives have in fact contributed not only to the material preservation of the Hyakugo Archive but also to the heritagization of both its material and immaterial being. In other words, they are two important moments in the "history of heritage" of the Toji Hyakugo Archive. Heritagization is not just a set of actions aimed at preserving an artifact but is part of the history of the artifact itself.

Similar to other examples of textual heritage, the Hyakugo Archive is particularly interesting because it forces us to rethink the relationship between the tangible and intangible aspects of heritage. All the intangible practices that have been carried out so far in relation to the Hyakugo Archive - cataloging, studying, digitizing, and creating "stories" about it - have been made possible by the existence of a tangible heritage - the archive itself - that has survived to this day thanks to a continuous process of re-evaluation, a reflection on the importance of preserving traces of the past as much as possible. Most importantly, it does not matter whether those who carry out these practices are able to read and enjoy every document in the collection - to carefully read tens of thousands of documents is a task that even Maeda Tsunanori could not have accomplished - the value of heritage is recognized for the Hyakugo Archive as a whole. It is correct to think of heritage primarily as an intangible cultural practice, as heritage studies show us, but none of these practices regarding the Hyakugo archive would be possible without tangible documents. As I have discussed elsewhere ^41^, texts can be interpreted as tangible embodiments of intangible practices, but at least in the case of textual heritage, intangible practice alone is insufficient to transmit such a large amount of information and knowledge over such a long period. We know that there are many examples of oral transmission of traditional knowledge - stories, songs, and crafts - that have survived exclusively through the intangible practice of storytelling based on the memory of living people, but no human mind could store such a large amount of information as we have in an archive: only texts can assure us this potential while at the same time shaping, with their "digital" fixity, the way we can recreate memories in our minds based on them.

## Conclusions

The digital revolution has finally freed text from its material and ephemeral embodiments, from the cages of paper, ink, and stone, making it possible to copy and share texts and words indefinitely and to store information in virtually infinite ways. This does not mean that textual heritage has become infinite or broader than before. To be called heritage, an object must be vividly present in people's minds. This must be an active part of the process of negotiating contemporary identities, languages, and cultures. An archive is an extremely intriguing example to reflect on heritage and heritagization because, unlike other kinds of heritage, such as monuments or dances, it is rarely fully read and enjoyed by the community that recognizes it as heritage. Very few people in Kyoto, including historians and archivists, have had the time and opportunity to read every document in the Hyakugo Archive, yet the people of Kyoto and Japan know its name and are proud to have a product of Japanese civilization inscribed in a UNESCO register. The Hyakugo Archive is a heritage site in Kyoto and Japan, even though much of it has never been displayed in exhibitions or public lectures. Every page included in the archive can become an occasion for new practices - translations, publications and exhibitions - that will strengthen its status as a cultural heritage.

Thus, the digitization of archives makes available to the public an impressive amount of data about manuscripts and artifacts of the past, theoretically enabling new heritage practices based on them. How all of this can benefit public awareness of museums, archives, and cultural products in general, and how this can be functional to UNESCO's goals as stated in the MoW Conventions - to promote understanding and human rights - is basically up to those who are committed to making all of this information present and alive in the public debate and in the shared knowledge of a community, be it local, national, or international. Given the importance that the "heritage paradigm" has enjoyed since the end of the last century, I would suggest that archival science in the 21st century should not ignore the ways in which its object of interest - manuscripts, documents, and archives - and the knowledge of how to read and manage them can become - or not - textual heritage.

## Ethics statement

Ethical approval and consent were not required.

## Data Availability

The data for this article consists of bibliographic references, which are included in the footnotes.
