# Supplementary material for: Textual heritage and digital archives – the case of the Hyakugo Archive in Kyoto
Source: Open Res Eur. 2023 Dec 20;3:218. [Version 1] doi: 10.12688/openreseurope.16820.1 (PMC10861945; doi:10.12688/openreseurope.16820.1)
Supplement: Peer review history

# Reviewer report: Reviewer response for version 1

**DOI:** 10.21956/openreseurope.18175.r37250

**Published:** 2024-02-12

**Authors:** Hideaki Akamatsu

**Affiliations:** 1 Faculty of Humanities Department of History and Cultural Properties, Beppu University, Beppu, Japan

This article explores the modern importance of archives as heritages, using the Toji Hyakugo Archive preserved at Toji Temple in Kyoto, Japan, as a specific example. This document was inscribed on UNESCO's Memory of the World list in 2015. The following is a brief summary of the article's contents.

 Introduction:

 In response to recent advancements in digital technology and discussions within critical heritage studies, Dr. Gerlini advocated for the examination of archives through a heritage perspective. He specifically highlighted the Toji Hyakugo Archive as the focal point of analysis.

 Main Discussion:

 Firstly, the article organized and presented an overview of discussions on the term "heritage" and various contemporary issues. Building upon Dr. Gerlini's discourse on textual heritage, the argument was made that the reproducibility of content is a characteristic defining archives as heritage. It was emphasized that heritage involves contemporary judgment and politics. The Toji Hyakugo Archive and preservation activities by Tsunanori Maeda were introduced, asserting that Maeda's actions represented an early instance of heritagization, encompassing not only the objects but also the activities of individuals. The example of *Seisatsu*, an order issued by Nobunaga Oda, a renowned figure in Japanese history, was cited to underscore the importance of such documents, even if they held limited historical value, due to their association with significant figures. Shifting to the present, the digitalization of historical document images and the sharing of Toji Hyakugo Archive-related stories on the "Hyakugo Archives WEB" were evaluated as initiatives contributing both to heritagization and preservation.

 Conclusion:

 The advancement of digital technology has enabled new approaches to archives. However, recognition as heritage is contingent upon historical and contemporary value being widely shared among many people. It is contemplated that the efforts of individuals involved in archives and historical documents are crucial in this regard.

 Reviewer's Perspective:

 The reviewer is a historian specializing in medieval Japanese history, and has elucidated the history of *Shōen *(estates) ruled by Toji through the analysis of the "Toji Hyakugo Archive. Japanese historiography, especially the study of Japanese history, has developed independently by incorporating Western historical influences in the late 19th century, while based on the ancient tradition of historical writing. While influenced by Western influences, geographical distance and language differences have allowed Japan to build its own academic community. From the standpoint of such a Japanese historian, I would like to add my comments on the value of this research.

 Evaluation Points for this Article:

1. The accomplishment of this article is that it focuses on Japanese archives, which do not receive much attention abroad, and discusses them based on the discussion of critical heritage studies, and clearly shows the value of Toji Hyakugo Archive as both tangible and intangible heritage.
2. For a historical researcher whose research theme is the content analysis of archival documents, the perspective of considering archives as heritage feels exceptionally innovative. From my own experiences, even when the Toji Hyakugo Archive was inscribed on UNESCO's Memory of the World Heritage list in 2015, I understood their global recognition, yet I had not conducted further in-depth analysis. In contemplating the contemporary value of archives that historical researchers study and how these archives can be positioned as heritage, I find this article to be insightful and commendable.

Improvement Points for this Article:

 While this article is highly valuable and requires no major revisions, attention should be paid to the following two minor points.

1. Archives are considered primary sources of information produced by various individuals and organizations regardless of time period or medium (Masahito Ando, 1998: "A challenge to archive science”), (Uejima, 2015, p.4-5). Human judgment is essential for the preservation of archives, but determining fair criteria for deciding whether or not to preserve them is an ongoing consideration. On the other hand, like archives, heritage are also selected by human judgment from tangible and intangible activities of the past. However, unlike the selection of archives, the selection of heritages is more subjective and involves political elements. Should researchers be more proactive than just studying and preserving archives? I would have liked to see further discussion on the role of the researcher with regard to heritagization.
2. Regarding the explanation of the Toji Hyakugo Archive (p. 9). It is a common understanding in current research on Japanese history that the *Shōen *(estate) system was established in the 11th century as an alternative social system to the *Ritsuryō* system, and that the *Ōnin* War in the late 15th century led to its collapse. The article states that the estate system started in the 8th century and disappeared under the Tokugawa Shogunate in the 17th century, which needs to be revised. I recommend reading (Toshikazu Ito, 2021) as an easily accessible and important reference.

If any, are all the source data and materials underlying the results available?

Yes

Is the work original in terms of material and argument?

Yes

Is the work clearly and cogently presented?

Yes

Is the argument persuasive and supported by evidence?

Yes

Does the research article contribute to the cultural, historical, social understanding of the field?

Yes

Does it sufficiently engage with relevant methodologies and secondary literature on the topic?

Yes

Reviewer Expertise:

History studies, particularly focusing on Japanese Medieval History

I confirm that I have read this submission and believe that I have an appropriate level of expertise to confirm that it is of an acceptable scientific standard.

# Reviewer report: Reviewer response for version 1

**DOI:** 10.21956/openreseurope.18175.r37256

**Published:** 2024-01-30

**Authors:** Titia van der Werf

**Affiliations:** 1 Research, OCLC BV (Ringgold ID: 58627), Leiden, South Holland, The Netherlands

Gerlini’s article is insightful and confusing at the same time. The author calls attention to textual sources, documents, and archives, that have been ignored by humanities scholars engaged in heritage studies. He proposes a definition for this missing category which he calls “textual heritage”. He elaborates on the characteristics of this category and argues that text is a digital innovation, because it can be easily reproduced and transferred to other carriers, without losing information or meaning. Gerlini proposes to reimagine text as a native digital product and recognize “textual heritage” as an “implicit aspect of digital heritage”. According to him, this new conceptualization of “textual heritage” has the potential to stimulate further reflections on the essence of heritage more broadly, and could fuel discussions on how texts, documents, and archives function as cultural heritage. He emphasizes that preserving texts does not automatically confer heritage status on them and explains how heritagization can become a long-term (political) process of valorisation. He proposes to examine “what issues arise during the process of heritagization in relation to archival sources”, on the basis of a case study of the Hyakugo archive. As the author demonstrates, this archive is a very good example of heritage, as evidenced by the long-term process of heritagization it has been undergoing in the past four centuries. Gerlini observes that the archive as-a-whole is considered heritage, even though it is rarely fully read. He also notes that digitization has allowed people to access this heritage, further amplifying the heritagization process and theoretically, enabling new heritage practices. The author concludes that “textual heritage”, such as the Hyakugo archive, allows scholars to reflect on heritage and heritagization in new ways. He specifically encourages archival science to look at archives through the lens of the new "heritage paradigm", in terms of how they can become - or not – “textual heritage”.

 This article gives an insightful overview of the concepts, main ideas and perspectives on cultural heritage from the subfield of critical heritage studies. The supporting references to relevant literature provide good starting points for the (non-expert) reader who wants to delve deeper into the subject. Coming from a libraries and archives background –where special collections and historical records are considered cultural heritage-, it surprises me to learn that these types of heritage are not studied in heritage studies. Drawing attention to this gap is a valuable contribution to advance research in this area. There are a few interesting interdisciplinary initiatives -like the critical archival and digital humanities studies cluster at the UCL’s Centre for Critical Heritage Studies–, worth mentioning in this context. Engaging archival scholars and digital preservation experts in critical cultural heritage research can bring fresh perspectives and lead to fruitful cross-pollination. As Gerlini suggests, it could trigger heritage custodians to think of their curatorial role as a form of intangible heritage and a function of heritagization. Cross-pollination would also help straighten out the use of terminologies across disciplines. This leads me to my next point.

 I find Gerlini’s reasoning for the need for a new category “textual heritage” confusing. The section titled “Text and archives as heritage” -in which the author explains why text is different from other content types and more similar to digital- is problematic because of its confusing use of terms and concepts, at least from the perspective of archival management and digital preservation. This section makes assertions that are debatable and raises questions and doubts about the argument made, leaving the reader unsure as to why processes of valorisation and meaning-making around archives can be better explained and understood through the theoretical category of "textual heritage". This section is up for debate.

 I would recommend discussing the category of documentary heritage more in-depth in the section on “UNESCO Memory of the World and the Documentary Heritage” and help the reader understand why this concept is not adequate for the purpose of this paper.

 The case study of the heritagization of the Hyakugo archive is perfectly understandable with the concept of documentary heritage and it is not obvious how talking about this archive as “textual heritage” makes the heritagization process more understandable. It would be very helpful if the author could clarify this.

 Besides addressing the above, there are a couple of other opportunities for improving the rigour and understandability of the article:

1. In the introduction, the author proposes to answer “some” of the questions listed, and in other sections, he proposes to answer additional questions. It would improve the reader’s understanding of the article’s intention, purpose and contribution to the field of research, if the research questions answered in this article were clearly defined, from the start. I recommend making the answers to the questions more explicit as well, relating them more clearly to the questions.
2. The article builds on previous work by the author, in which the concept of “textual heritage” has been used before. Because this concept is central to this article and its argumentation, I recommend providing a short overview explaining how this article relates to previous own work and what this article is adding to it.  

The suggested clarifications and additions should make the article more robust and better equipped to engage with scholars from archival sciences, digital humanities, digital preservation, and cultural heritage studies on the research questions it addresses. I look forward to this conversation!

If any, are all the source data and materials underlying the results available?

No source data required

Is the work original in terms of material and argument?

Yes

Is the work clearly and cogently presented?

Partly

Is the argument persuasive and supported by evidence?

Partly

Does the research article contribute to the cultural, historical, social understanding of the field?

Partly

Does it sufficiently engage with relevant methodologies and secondary literature on the topic?

Yes

Reviewer Expertise:

Digital libraries, digital preservation, archival management

I confirm that I have read this submission and believe that I have an appropriate level of expertise to confirm that it is of an acceptable scientific standard, however I have significant reservations, as outlined above.

# Response

**Published:** 2024-01-30

**Authors:** Edoardo GERLINI

Thank you very much for your review. The points you raised are entirely agreeable, particularly concerning the lack of clarity in defining theoretical terms such as "text" and "heritage." I will strive to be more convincing in the textual heritage section, providing better clarification for why the category of tangible heritage alone is not sufficient to appropriately describe the historical and cultural processes that have developed around historical archives. It's true that the various questions I raise in the introduction might confuse the reader about what the article is really about. However, since the primary goal of the article is to offer an interdisciplinary and (possibly!) innovative view, I thought it would be helpful to present a range of questions that other scholars might find interesting. One solution could be to move these unanswered questions to the end of the article...? In this way, the introduction will be more aligned with the rest of the article, while in the conclusions, I would address the comment from the reviewer who asked for examples of how archival studies and heritage studies can collaborate I will also add a very short description of how I applied the concept of textual heritage in my previous works.

# Reviewer report: Reviewer response for version 1

**DOI:** 10.21956/openreseurope.18175.r37257

**Published:** 2024-01-17

**Authors:** David Harvey

**Affiliations:** 1 School of Culture and Society - Department of Archeology and Heritage Studies, Aarhus University, Aarhus, Denmark

This is a well written article which explores the relationship between 'heritage' and 'text', specifically with respect to seeing 'digital archives' as 'heritage' - whether digital archives can be considered 'heritage', and (if so) what the implications of this are, both for critical heritage studies digital textual studies. In this respect, it speaks to two of the key themes (trends?) that humanities scholars are interested in right now: 'heritage' and the 'digital humanities'!

 The basic issues and questions are set out clearly in the introductory sections, which also sets out some interesting links to issues of human rights - the right to cultural life - which is how the author gain traction on the subject matter through the Faro Convention (on cultural heritage and society), and the Memory of the World (MoW) digital collection. On first glance, I pondered whether some clearer definitions (of 'heritage' and 'text') would have been more usefully laid out earlier, but in practice, I think the paper works well through a slightly longer meditation on/unpacking of the terms in a manner that highlights the gaps and points to the opportunities - as to what thinking about (digital) 'texts' does for critical heritage studies, and what processual ideas of heritage does for thinking about digital texts. The wider literature cited all makes sense, but I did wonder whether the author would benefit from looking at the work of Liz Stainforth:

 Stainforth, E (2020) 'Distruptive forms, persistent values: negotiating digital heritage and "the Memory of the World"', in T. Carter et al *Creating Heritage: Unrecognised Pasts and Rejected Futures, *(Routledge) p. 32-45. Or, Grincheva, N and Stainforth, E. (2023) *The Geopolitics of Digital Heritage, *(CUP)

 I don't know much about the Hyakugo archive (or the wider topic of Japanese literature), but I can vouch for this paper being clear to a non-expert reader who is approaching this material for the first time. It is really interesting to see how my own previous work on the 'history of heritage' has encouraged these types of study, which explore the potential of this 'historical-biographical' approach. Indeed, the author might find Chris Siwicki's work useful on this - he looked at Roman ideas of (something that might be called) 'heritage' ( *Architectural Restoration and Heritage in Imperial Rome*, OUP, 2019).

 The conclusions are brief - perhaps being a bit more clear (and developed) on how work on 'critical heritage' and 'digital texts' can be mutually enabling, might be good? Indeed, I think the last line is a bit tantalising and also (perhaps) negative - rather than suggesting that archival science 'should not ignore' the field of 'textual heritage', perhaps the author can lay out more confidently, what such a reflection might enable... how those interested in digital archives (and those working in critical heritage) have something to gain, by thinking about 'textual heritage'.

If any, are all the source data and materials underlying the results available?

Yes

Is the work original in terms of material and argument?

Yes

Is the work clearly and cogently presented?

Yes

Is the argument persuasive and supported by evidence?

Yes

Does the research article contribute to the cultural, historical, social understanding of the field?

Yes

Does it sufficiently engage with relevant methodologies and secondary literature on the topic?

Yes

Reviewer Expertise:

Heritage studies, historical and cultural geography.

I confirm that I have read this submission and believe that I have an appropriate level of expertise to confirm that it is of an acceptable scientific standard.

## References

[1] : Distruptive forms, persistent vlaues: negotiating digital heritage and "the Memory of the World dust. *https://eprints.whiterose.ac.uk/126992/13/Stainforth-DigitalHeritage.pdf* .2020;

[2] : Creating Heritage: Unrecognised Pasts and Rejected Futures dust. *Routledge* .2020;

[3] : Geopolitics of Digital Heritage dust. *Cambridge University Press* .2024;

[4] : Architectural Restoration and Heritage in Imperial Rome.2019; 10.1093/oso/9780198848578.001.0001 10.1093/oso/9780198848578.001.0001

# Response

**Published:** 2024-01-30

**Authors:** Edoardo GERLINI

Thank you very much for the encouraging comment and the useful suggestions you gave me. I agree that the way I exposed slippery concepts like "text" and "digital" may result too shallow and somehow imprecise, but because these terms underwent so many interpretations, I tried to keep my assertion simple and straightforward. I will try to improve this part. The references you suggested me will sure make my argument more reliable. Thank you very much. I will also expand the conclusions too, even if speaking from the standpoint of a philologist in the so called area studies, I am not so much credited to speak for archivists or heritage scholars.
